# Liquid Biopsy to Detect DNA/RNA Based Markers of Small DNA Oncogenic Viruses for Prostate Cancer Diagnosis, Prognosis, and Prediction

**DOI:** 10.3389/fonc.2020.00778

**Published:** 2020-07-08

**Authors:** Maurizio Provenzano, Abdou Kamal Allayeh

**Affiliations:** ^1^Oncology Research Unit, Department of Urology and Division of Surgical Research, University Hospital of Zurich, Schlieren, Switzerland; ^2^Department of Immunology, University Hospital of Zurich, Zürich, Switzerland; ^3^Virology Lab 176, Environmental Research Division, National Research Centre, Cairo, Egypt

**Keywords:** prostate cancer, liquid biopsy, small DNA viruses, polyomavirus, large T antigen

## Introduction

The National Cancer Institute (NCI) defines a non-solid or liquid biological biopsy as follows: a test done on a sample of blood to look for cancer cells from a tumor that is circulating in the blood or for pieces of DNA from tumor cells that are in the blood. In short, any liquid specimen can be used to easily study the onset, progression, and relapse of cancer. Although mostly used for cancer diagnosis/prognosis, liquid specimens can be involved in several different fields, such as virus detection. Presently, the association between cancer and viruses is under continuous investigation, as ~20% of human cancers worldwide have an infectious etiology, mainly viruses (1).

Prostate cancer (PCa) is the most common cancer in men accounting for 47.2% of all new cancer cases and is the fifth leading cause of male cancer mortality (6.6%) worldwide by geographic areas (2). PCa is a slow-growing, organ-confined tumor usually accompanied by a favorable overall prognosis. However, a large proportion of treated PCa eventually develops into a hormone-independent disease that often progresses to metastatic castration-resistant prostate cancer (mCRPCa) (3). As such, the search for biomarkers to precisely and non-invasively characterize the biology of PCa is of utmost importance to researchers. However, the quest for a single PCa marker that can identify patients with an early aggressive or a clinically significant disease and determine their prognosis is still ongoing (4).

High throughput technologies, such as quantitative real-time PCR (qRT-PCR), Tissue Microarray (TMA)—Fluorescent *in situ* hybridization (FISH), and DNA microarray, have enhanced the detection of genetic signatures in cancer specimens. To date, several biomarkers have been evaluated for their use in PCa diagnosis, prognosis, and therapeutic interventions. However, validation studies are needed in order to implement them as clinically useful markers. In addition, their role in PCa onset and progression remain elusive. The biological tissues mainly used for the identification of PCa biomarkers are solid tissues from biopsies/tumors or non-solid tissue, such as blood. Although efficient, the techniques used to obtain these samples are very invasive. Recently, urine has emerged as a non-solid biological tissue for suitable and affordable screening tests (5).

Indeed, DNA/RNA-based markers, which are mainly detectable in the urine of PCa patients, show promise. The association between infectious agents and PCa, as suggested for small DNA viruses, is still strongly debated. Here, we provide the readers with a general overview about suitable and feasible technologies to use specifically for PCa diagnosis/prognosis. Therefore, the state-of-the-art of these procedures might be relevant to understand the modalities in use to detect small DNA viruses in this malignancy.

### Standard Diagnostic Procedure

Currently, the selection of patients at risk of PCa is based on a combination of blood testing for the prostate specific antigen (PSA), an enzyme produced by the prostate in either physiological or pathological conditions, and the digital rectal examination (DRE) of the organ (PSA+DRE diagnostic procedure) (6). Indeed, a PSA level >4 ng/mL and a positive DRE are sufficient ground for patients at risk to undergo prostate biopsy, although the European Randomized Study of Screening for Prostate Cancer (ERSPC) and the Prostate, Lung, Colorectal, and Ovarian (PLCO) Cancer Screening Trial do not attribute a survival benefit in detecting PCa in case of suspicious DRE (7, 8).

Approximately 19 million men in the USA are currently screened annually with PSA blood tests for PCa. The total market for PCa diagnostics is significant, with an estimated 14.3 billion USD turnover in 2017 at an annual growth rate of 7.5%. Therefore, improving current methods for screening of PCa patients is of great importance and will positively affect a significant part of the population (8).

The refinement of current diagnostic procedures has decreased the mortality-to-incidence ratio of PCa by identifying early-stage PCa (low grade prostatic intraepithelial neoplasia; LG-PIN) which can either be monitored (active surveillance) or immediately treated (prostatectomy). Nevertheless, the limitation of PSA as a diagnostic marker is due to an elevated negative biopsy rate (75% of false positive patients), whereas the repetition of the first negative biopsy does not always lead to better results. Hence, only 25% of patients who undergo a prostate biopsy upon selection based on suspicious DRE and borderline PSA test (2.5/4.0 and 10.0 ng/ml) show the presence of a tumor. In addition, acting within this PSA “gray zone” may result in not only an elevated negative biopsy rate but also in a chance of ~25% of missing tumors (9). Therefore, using PSA as a diagnostic marker leads to over diagnosis, a major concern of the PSA+DRE diagnostic procedure.

Finally, the main limitation of the PSA+DRE diagnostic procedure is the inability to distinguish clinically significant cancers (pathologic stage, tumor volume, and cancer grade) from non-life-threatening tumor lesions in the prostate. Therefore, the PSA blood test, despite being the golden standard for PCa active surveillance and recurrence, has several limitations regarding screening patients at risk of PCa.

### Novel Screening Procedures for PCa Diagnosis/Prognosis by Urine Test

Thus far, diagnostic/prognostic markers have been studied in human specimens, such as cancer tissues or blood obtained through surgery/biopsy or venipuncture procedures. Although efficient, they are invasive, creating pain and discomfort in patients (rounds of core biopsies) and as such cannot be performed routinely. In order to avoid that, the selected specimen should be collected by non-invasive methods, as is the case with urine. Recently, gene-based markers that are mainly detected in urine have shown promise. In 2012, the FDA approved a PCa test based on the detection of prostate cancer gene 3 (PCA3) in urine (10).

PCA3 is a non-coding mRNA detected in the urine of patients after DRE/prostate massage (11). Transcription-mediated amplification technology is used to amplify the mRNA molecules of the prostate cells collected in the sample. Normally, the result is reported as the ratio of PCA3 mRNA to PSA mRNA (PCA3/PSA). Higher PCA3/PSA ratios are an indication of PCa risk (12). Although PCA3 is restricted to the prostate, as opposed to PSA, the physiological or pathological role of PCA3 in the homeostasis of the prostate or in disease development is largely unknown. In addition, PCA3 cannot independently predict prognosis in PCa patients (i.e., the biochemical recurrence) as PSA does. However, PCA3 plays a role in the risk classification of patients undergoing active surveillance (13).

Another promising urinary biomarker test is encoded by a fusion gene formed as a result of a translation between the androgen-regulated trans-membrane protease, serine 2 (TMPRSS2) gene transcriptional promoter and the ETS-related oncogene (ERG), resulting in an androgen-regulated TMPRSS2-ERG fusion gene that is highly PCa-specific (14). The transcripts of TMPRSS2-ERG fusion were analyzed in urinary sediments and showed sensitivity of 37 and 93% of specificity for PCa prediction (15). In addition, TMPRSS2-ERG fusion was associated with pathological stage (16), Gleason score (17, 18), and with PCa death (16). Additional biomarker could further boost sensitivity and specificity in a multiplex detection system.

As DNA methylation play a role in the onset/progression of several carcinomas (19, 20), including PCa (21), for completeness of information, reports that are focused on the urinary DNA methylation biomarkers in PCa (22, 23) are here also added.

### Advantages and Disadvantages of Using Liquid Biopsies

Despite liquid biopsies being the new frontier for cancer diagnosis and prognosis, there are several key points that need to be addressed. First, when using new specimens, it is necessary to detect the presence of the marker of choice in the selected specimen at high quality and quantity levels. Secondly, the marker needs to be detected without adopting extra procedures (24).

Not all types of specimens perform better than all conventional tests (i.e., ELISA, qRT-PCR). For example, gene expression analysis of a specific marker may not be confirmed at a protein level when using the same specimen. As such, different methodologies may be more appropriate depending on the type of substrate used. In that case, the better choice would be using a single high throughput technology or changing the parameters to adjust marker activity by using other procedures. PSA is a marker which is detectable in blood, but it is quite difficult to detect its mRNA directly in urine. Patients tested with PSA need to undergo either DRE or prostate massage to increase the number of prostate cells, mainly cancer cells, in urine, which can improve PSA-mRNA detection (25). These procedures must also be performed when testing the non-coding mRNA of PCA3. Therefore, these tests might need to include uncomfortable procedures that will reduce the affordability and accessibility of the test (26).

### DNA/RNA-Based Markers and Their Diagnostic/Prognostic Potential

Detecting circulating tumor DNA (ctDNA) and micro-RNAs (mi-RNAs) in liquid biopsies can be used to assist in the clinical management of difficult-to-diagnose patients with advanced-stage cancers (27) and can be used partially for PCa diagnosis at early stage and prediction of pre-treatment prognosis. Circulating tumor DNA, which is detectable mainly in blood, has been considered a more suitable biomarker for advanced PCa. A recent study conducted in Sweden, which investigated the genomic landscape of metastatic PCa, has demonstrated the utility of ctDNA in PCa as a predictive marker for mCRPCa (28). As such, ctDNA in PCa is more suitable for biomarker-direct therapy than as a diagnostic tool (29). As detecting ctDNAs, microRNAs as well as viral DNAs/miRNAs in liquid biopsies by qRT-PCR and their DNA/RNA-based diagnostic/prognostic potential can be used partially for PCa diagnosis at early stage, the novel high throughput droplet digital PCR (ddPCR) has been employed to detect DNA/RNA-based markers in a variety of different disease, including cancer. For instance, ddPCR has been employed to detect circulating miRNA in sera from lung cancer patients (30). Similarly, this highly sensitive technique could be very helpful in detecting ctDNAs, microRNAs, and/or viral DNAs/miRNAs in liquid biopsies for PCa diagnostic purposes (31, 32). A recent study on ctDNA isolated from enriched CTC from surgically treated PCa patients undergoing androgen deprivation therapy showed a significant association between an androgen receptor transcriptional variant (V7-AR) detected by ddPCR, and a high probability of developing castration-resistant (CRPCa) and (mCRPCa) metastatic castration resistant PCa (33).

We have recently evaluated the impact of the techniques used to identify polyomavirus BK (BKPyV) DNA in cancer patients' specimens (34). Although limited for the purpose of the meta-analysis, it is evident that gene expression analysis by quantitative real-time PCR (qRT-PCR) is superior to immunohistochemistry (IHC). While IHC could be more useful when dealing with paraffin-embedded tissue specimens, gene expression can be used in samples ranging from solid-tissues to non-solid/liquid biological biopsies. Indeed, serum, plasma, and urine are the most used specimens for evaluating gene expression. As such, the lack of fresh specimens makes liquid biopsies the best choice. Circulating small DNA viruses (viremia) are not particularly useful in evaluating virus-induced oncogenesis in human organs, including the prostate. Most small oncogenic DNA viruses are latent viruses. Therefore, their reactivation is often induced by the dysregulation of the balance between viruses and the immune system (35).

Although a higher concentration of viruses could be correlated with a higher chance of tumor transformation, the oncogenic activity of small DNA viruses always involves an abortive infection, which is an uncoupling of early gene expression from late gene expression, thus inhibiting viral assembly and virion formation which normally occurs after a permissive infection (36). In addition, cancer patients at early stages hold an immunocompetent status, which is in contrast with a latent life-threading virus reactivation (37). Therefore, a negative viremia is normally expected (cut-off >4log geq/mL). However, a weak viruria could be detected (cut-off >7log geq/mL) (38).

The latter is due to the virus spreading in the urine because of a smoldering infection. Therefore, viruria can indirectly offer information about the status of the immune system in patients, as BKPyV reactivation occurs mainly in immune-compromised subjects. Indeed, Tag IgG serology could be used as a prognostic factor for PCa (39).

Several human miRNAs have been shown to be dysregulated in PCa and to influence PCa onset and progression by regulating key cancer genes. Therefore, the detection of circulating miRNAs in the body fluids of PCa patients is one of the major diagnostic tests for this disease. Some of these miRNAs can discriminate between the presence and absence of cancer with diagnostic accuracy and especially predict prognosis (miR-16, miR-26a, and miR-195) (40).

In recurrent PCa, miR-1 was significantly down-regulated, compared to non-recurrent PCa (*P* < 0.001). The study included 78 patients; 27 recurrent PCa and 51 non-recurrent PCa. The study of Cox proportional hazards showed that miR-1 might be an independent prognostic factor for the recurrence of PCa (HR: 1.86, 95% CI: 1.21–2.94; *P* = 0.011) (41). The human miR-129 was isolated from peripheral blood mononuclear cells of 98 PCa patients and significantly down-regulated, compared to 56 controls (*P* < 0.05) (42). The expression levels of miR-21 was significantly associated with Gleason level, tumor stage, bone metastasis, and recurrence (*P* < 0.05), as relative to benign control group (*P* < 0.05). The results suggest the expression rate of miR-21 as strong biomarker for PCa prognosis (43).

Moreover, seven miRNAs (let-7c, let-7e, let-7i, miR-26a-5p, miR-26b-5p, miR-18b-5p, and miR-25-3p) were found in the serum of PCa patients and could differentiate PCa from BPH (44). Also, miR-191 and let-7 family were identified in urinary sediments of PCa, which could use as non-invasive biomarkers in the diagnosis of PCa (45, 46). The role of circulating miR-141 and miR-375 as potential biomarkers for PCa progression, as previously validated in murine models, was confirmed by analyzing the quantitative differences in their gene expression in the sera of PCa patients vs. benign prostatic hyperplasia (BPH) controls, as well as PCa patients with or without recurrence (47) and mCRPCa patients vs. low risk localized PCa (48). The same authors confirmed the role of urine as a relevant specimen for biomarkers detection (47).

Liquid biopsies were also used to test the expression profile of miR-141 as a predictive marker for PCa (49). Based on these reports, both miRNAs are good candidates for biomarkers that can be used for PCa diagnosis and disease outcome. It is important to emphasize that circulating miRNAs are associated with risk evaluation by the UCSF-CAPRA score (50). For completeness of information about circulating miRNAs and PCa, the following miRNA are also added: miR-221 and miR-21 (40); miR-628-5p, miR-101, and miR-25 (51); miR-1825, miR-484, and miR-205 (52).

A meta-analysis has recently showed an association between herpes virus infections and the increasing risk of PCa development. Indeed, Herpes-miRNAs (hsv1-miR-H18and hsv2-miR-H9-5p) have been detected in urine of PCa patients and can be used as diagnostic biomarkers for this malignancy (53). Specifically, in urine samples of PCa patients, the expression levels of hsa-miR-615-3p, hsv1-miR-H18, hsv2-miR-H9-5p, and hsa-miR-4316 were significantly higher than in BPH controls. In general, hsv1-miR-H18 and hsv2-miR-H9-5p derived from herpes simplex virus showed better diagnostic performance than PSA test for patients in the PSA gray zone.

In addition, a combination of urinary hsv2-miR-H9-5p with serum PSA demonstrated high sensitivity and specificity, providing a potential clinical benefit by minimizing unnecessary biopsies. In another study (54) conducted on graft biopsies and urine samples collected from kidney transplant recipients, BK polyomavirus miRNAs were found to be highly abundant in the fraction of urinary exosomal enrichment in BK nephropathy patients. This study suggested that urinary exosomal bkv-miR-B1-5p and bkv-miR-B1-5p/miR-16 could be surrogate markers for the diagnosis of BK polyomavirus nephropathy in kidney transplant recipients.

Finally, polyomavirus circulating miRNAs have shown potential involvement in cancer transformation, as reported for SV40 miRNA (55, 56). Imperiale and his colleagues first described the role of polyomavirus JC (JCPyV) and BKPyV miRNAs in regulating the expression of the regulatory protein Tag (by the degradation of the mRNA from the Tag ORF) (57, 58). The viral miRNA, which functions as a regulator of host or viral gene expression (59) might modulate Tag-specific immune activity (57). As such, BKPyV-Tag negative patients could bear cognate memory T cells. In this case, this high-throughput method should be efficient enough to permit selection of neutralizing antibodies from quiescent Tag memory B cells. The potential oncogenic role of polyomavirus circulating miRNAs could be extended to Merkel Cell polyomavirus (MCPyV) miRNA (60) and SV40 miRNA, although their association with PCa development is no longer a subject of investigation, unless for SV40 transgenic mice (TRAMP mice) (61).

## Conclusion

Prostate cancer is common, influencing practically 50% of men more than 50 years old. Advanced rectal assessments and blood tests for PSA are the two most regular demonstrative diagnostic tools. Although the PSA is useful, there are some limitations. The NCI gives a model; about 25% of men who have a prostate biopsy because of a high PSA level are found to have prostate malignant growth. For this reason and others, scientists are exploring different methods for checking of PCa, and some are looking to the urine. As fluid goes through the urethra from the prostate, it brings cellular and molecular markers/wastes with it, such as cancer cells and microRNAs. Researchers can utilize it to discover hints about the presence of PCa once the body has passed these biomarkers out in the urine. Contrasted with other biological fluids, urine has the benefits of being inexpensive, metabolites-rich, easy to handle, and usable in enormous quantities, without needing obtrusive collection treatments. However, it is described by low concentrations of potential biomarkers and high varieties among patients relying upon sex, age, physical action, or disease stage.

Several studies have correlated small DNA viruses with prostate cancer, suggesting that these viruses exhibit oncogenic activity in the early stages of cancer development. However, the mechanism responsible for the implication of small DNA viruses in cancer development remains unclear. For urinary viral infections encoded microRNAs, particularly herpes virus and Bk polyomavirus, could be used as significant diagnostic biomarkers in PCa development. Not only herpes or Bk polyomavirus infections, but also all viruses encoded microRNAs such as Epstein-Barr virus, other polyomaviruses, and papillomaviruses require further studies and more laboratory tests to determine the role of their circulating miRNAs in early detection or development and progression of cancers using urine sample. At the end, identifying potential candidates for the signature of circulating human or viral miRNAs in urine samples would have significant implications for an alternative or supportive diagnostic tool. To the best of our knowledge, all techniques discussed in this opinion article are the only adopted by laboratories to detect small DNA viruses in PCa (i.e., miRNA) and the great opportunities offered by liquid biopsies, such as urine, to detect small DNA viruses are yet to be fully explored.

## Author Contributions

MP conceived and wrote the paper; AA wrote the paper.

## Conflict of Interest

The authors declare that the research was conducted in the absence of any commercial or financial relationships that could be construed as a potential conflict of interest.
